# Antibody levels against GLURP R2, MSP1 block 2 hybrid and AS202.11 and the risk of malaria in children living in hyperendemic (Burkina Faso) and hypo-endemic (Ghana) areas

**DOI:** 10.1186/s12936-016-1146-4

**Published:** 2016-02-27

**Authors:** Bright Adu, Mariama K. Cherif, Samuel Bosomprah, Amidou Diarra, Fareed K. N. Arthur, Emmanuel K. Dickson, Giampietro Corradin, David R. Cavanagh, Michael Theisen, Sodiomon B. Sirima, Issa Nebie, Daniel Dodoo

**Affiliations:** Noguchi Memorial Institute for Medical Research, University of Ghana, Legon, Ghana; Polytechnic University of BoboDioulasso, Bobo-Dioulasso, Burkina Faso; Centre National de Recherche et de Formation sur le paludisme, Ouagadougou, Burkina Faso; School of Public Health, University of Ghana, Legon, Ghana; Department of Biochemistry and Biotechnology, Kwame Nkrumah University of Science and Technology, Kumasi, Ghana; Department of Biochemistry, University of Lausanne, Epalinges, Switzerland; Institute of Cell, Animal and Population Biology, School of Biological Sciences, University of Edinburgh, Edinburgh, Scotland, UK; Department for Congenital Disorders, Statens Serum Institut, Copenhagen, Denmark

**Keywords:** Malaria, Antibodies, GLURP R2, MSP1 block 2 hybrid, AS202.11, Hyperendemic, Hypo-endemic, Transmission intensity

## Abstract

**Background:**

Differences in parasite transmission intensity influence the process of acquisition of host immunity to *Plasmodium falciparum* malaria and ultimately, the rate of malaria related morbidity and mortality. Potential vaccines being designed to complement current intervention efforts therefore need to be evaluated against different malaria endemicity backgrounds.

**Methods:**

The associations between antibody responses to the chimeric merozoite surface protein 1 block 2 hybrid (MSP1 hybrid), glutamate-rich protein region 2 (GLURP R2) and the peptide AS202.11, and the risk of malaria were assessed in children living in malaria hyperendemic (Burkina Faso, n = 354) and hypo-endemic (Ghana, n = 209) areas. Using the same reagent lots and standardized protocols for both study sites, immunoglobulin (Ig) M, IgG and IgG sub-class levels to each antigen were measured by ELISA in plasma from the children (aged 6–72 months). Associations between antibody levels and risk of malaria were assessed using Cox regression models adjusting for covariates.

**Results:**

There was a significant association between GLURP R2 IgG3 and reduced risk of malaria after adjusting age of children in both the Burkinabe (hazard ratio 0.82; 95 % CI 0.74–0.91, *p* < 0.0001) and the Ghanaian (HR 0.48; 95 % CI 0.25–0.91, *p* = 0.02) cohorts. MSP1 hybrid IgM was associated (HR 0.85; 95 % CI 0.73–0.98, *p* = 0.02) with reduced risk of malaria in Burkina Faso cohort while IgG against AS202.11 in the Ghanaian children was associated with increased risk of malaria (HR 1.29; 95 % CI 1.01–1.65, *p* = 0.04).

**Conclusion:**

These findings support further development of GLURP R2 and MSP1 block 2 hybrid, perhaps as a fusion vaccine antigen targeting malaria blood stage that can be deployed in areas of varying transmission intensity.

**Electronic supplementary material:**

The online version of this article (doi:10.1186/s12936-016-1146-4) contains supplementary material, which is available to authorized users.

## Background

Falciparum malaria remains a significant cause of infant mortality and morbidity in many parts of the world especially in sub-Saharan Africa even though decreasing trends of transmission intensity have been reported [1–3]. An efficacious blood stage vaccine against malaria that remains potent in different transmission settings would greatly contribute to reducing the disease burden among endemic populations. However, the putative antigenic targets of protective immunity against malaria have remained elusive and several parasite proteins have been implicated, most of which are polymorphic. Antibody responses targeting polymorphic malaria parasite surface proteins have been associated with reduced risk of malaria [4–6]. This may partly explain the need for repeated infections in the acquisition of natural protective immunity among adults living in endemic populations [7]. While polymorphic antigens may not be the most ‘attractive’ candidates for vaccine design, it is thought that such a vaccine, if successful, could elicit a broad spectrum of immune repertoire particularly in children and other vulnerable groups [8]. The merozoite surface protein (MSP)1 is synthesized in a precursor form as a 195kD protein and proteolytically cleaved into fragments prior to schizont rupture [9]. The carboxy-terminal portion is largely conserved with only a few single nucleotide polymorphisms (SNPs) [10] while the amino-terminal portion has the most polymorphic region, termed block 2 [11, 12]. Antibody responses directed against the block 2 region have been associated with reduced risk of clinical malaria in different populations [5, 13, 14]. A synthetic MSP1 block 2 construct, based on several polymorphic variants found in natural *Plasmodium falciparum* isolates was designed and fused with the relatively conserved block 1 sequence of MSP1 to form the MSP1 block 2 hybrid [8]. This synthetic protein was immunogenic in experimental animal models and was recognized by sera from Burkinabe and Ghanaian children naturally exposed to the parasite [8]; however, studies assessing anti-MSP1 block 2 hybrid antibodies in relation to the risk of malaria in longitudinal cohorts is currently lacking.

The glutamate rich protein-region 2 (GLURP R2) is from the carboxy-terminal repeat region of GLURP and is the most immunodominant portion of the protein [15]. Compared to the amino terminal GLURP R0 region, which has been extensively studied [16, 17] and forms part of the GMZ2 candidate vaccine [18] presently in phase 2b clinical trials, GLURP R2 has been less studied. GLURP R2 contains at least two B cell epitopes and elicits antibodies capable of inhibiting malaria parasite growth in vitro in cooperation with monocytes [19]. Importantly, anti-GLURP R2 antibodies were associated with reduced risk of symptomatic malaria infection in Burkinabe [20] and Ghanaian [21] children. Alpha (α) helical coiled motifs in malaria antigens, such as MSP3 and MSP6, are important oligomerization sub-units and targets of malaria protective antibodies [22, 23]. When separated from the whole protein, α-helical coiled motifs readily fold into the same stable oligomeric structure [24]. Thus, such motifs could potentially be fused to other antigenic targets of malaria protective antibodies to form chimeric proteins capable of eliciting broader spectrum immune response. The peptide AS202.11 (PF11 0424) (described elsewhere [25]) is an α-helical coiled motif. Antibody responses to this peptide showed a modest association with reduced risk of clinical malaria in children resident in the Kilifi district of Kenya [25]. This study successfully evaluated the associations between antibody responses against GLURP R2, MSP1 block 2 hybrid and the peptide AS202.11 and the risk of malaria in two populations (Burkina Faso and Ghana) with different malaria transmission intensities.

## Methods

### Ethics statement

The Burkina Faso study was approved by the Ethical Committee for Biomedical Research of the Ministry of Health of Burkina Faso, while in Ghana, the study was approved by the Institutional Review Board of Noguchi Memorial Institute for Medical Research (NMIMR) of the University of Ghana, Accra. At both study sites, written informed consent was given by the parent or guardian of children prior to their enrolment into the study.

### Study sites

#### Burkina Faso: Balonghin–Sapone

The Sapone health district is 50 km southeast of Ouagadougou, the capital city of Burkina Faso. The area has been described elsewhere [26]. The climate in this area is characteristic of the Sudanese savannah, with a dry season from November to May (low transmission season) and a rainy season from June to October (high transmission season). Malaria transmission is markedly seasonal; most transmission occurs during the rainy season. The entomological inoculation rate (EIR) in Balonghin was estimated at 0.3 infective bites per person per month during the dry season and 44.4 infective bites per person per month during the rainy seasons [26]. *Plasmodium falciparum* is the predominant malaria parasite, accounting for more than 95 % of infections.

#### Ghana: Asutsuare–Damgbe West

The study was conducted in Asutsuare about 120 km northeast of Accra and five neighbouring villages of the Damgbe West district in the southeastern part of Ghana, described elsewhere [27]. Briefly, rainfall is usually continuous throughout the year but highest from June to August and moderate in November and December just before the beginning of the dry season. Thus, there are two seasonal peaks of malaria transmission corresponding to the wet seasons but also relatively minimal transmission throughout the remaining times of the year. Malaria incidence in Asutsuare was 8.9 % in 2009 [27], the year of the present study. However, the incidence rate of clinical malaria was reported as 106.6 per 1000 population in Dodowa a suburb of about 44 km away in 1992 [28]. About 98 % of malaria cases in the area are due to *P. falciparum* infection while *Plasmodium ovale* and *Plasmodium malariae* account for the remaining [29].

### Study design, sample collection and follow-up

**Fig. 1:**
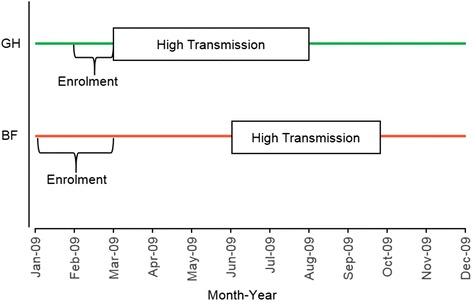
Site-specific transmission seasons and period of cohort enrolment. *BF* Burkina Faso, *GH* Ghana

In Burkina Faso, 525 children aged 6–60 months inclusive were enrolled in the study while the Ghanaian cohort consisted of 600 individuals aged one to 29 years. However, for comparison purposes, the current study excluded the older aged Ghanaians and only included 209 Ghanaian children (aged 12–72 months) and the 354 Burkinabe children who completed the follow-up. At both study sites, participants were enrolled prior to the high malaria transmission season (Fig. 1). During baseline (enrolment) sampling at each site, 5 ml (or about 1 ml for younger children) EDTA-anticoagulated venous blood was collected from each child and centrifuged. Plasma obtained was aliquoted and stored at −20 °C for immunological analyses. Thick and thin film blood slides (TTBS) were obtained for microscopy diagnosis of malaria. The ensuing active case detection during the longitudinal follow-up was either twice weekly (Burkina Faso) or weekly (Ghana). The follow-up duration was 39 weeks for the Burkinabe and 36 weeks for the Ghanaian cohorts, respectively. Axillary temperature was measured on each visit of a child by a trained field assistant and children with fever, defined as axillary temperature >37.5 °C or a history of fever reported within the last 24 h. In Burkina Faso, malaria rapid diagnostic test (RDT) was performed and children with positive results were referred to the nearest health centre for appropriate treatment. In Ghana, children with fever were referred without RDT, to the health centres and TTBS made. Children diagnosed with malaria were treated with artemisinin-based combination therapy which was the standard treatment for malaria in both countries at the time of the study. At both study sites, clinical malaria was defined as axillary temperature ≥37.5 °C (or history of fever in the last 24 h preceding the visit) and parasite density ≥5000/μl with at least one other sign of malaria such as vomiting, diarrhoea or malaise. Parasite density determination by microscopy was determined as previously described [26].

### Malaria antigens

The recombinant GLURP R2 (amino acids 705-1178) of the carboxy-terminal repeat region expressed in *Escherichia coli* [15]. The MSP1 hybrid (described in detail elsewhere, [8]) was from a synthetic gene designed to incorporate sequences derived from one version of the semi-conserved block 1 and all three serotypes of the highly polymorphic block 2 of MSP1. Briefly, the upstream sequence corresponds to the block 1 sequence from K1-type MSP1 and is followed by artificially designed sequences which include the three main variants of the block 2 found in over 150 laboratory and field isolates. The RO33 type contains minimal point mutations only while the other two types (K1 and MAD20) are characterized by unique, serotype-specific flanking sequences, each containing different sets of tripeptide repeats which differ within each serotype by both order and number. The resulting MSP-1 block 2 hybrid construct of 348 amino acids in length (in a pET24a expression vector) was expressed as a 31 kDa untagged protein in the *E. coli* BLR (DE3) pLysS strain. The synthetic peptide, AS202.11-(QLEEKTKQYNDLQNNMKTIKEQNEHLKNKFQSMGK) used in the study has been described in detail elsewhere [30].

### Antigen-specific antibody measurement

Antigen-specific antibody levels were measured using the Afro Immuno Assay (AIA) ELISA protocol [16, 31] with modifications. Briefly, 96-well microtitre ELISA plates (Maxisorp Nunc, Denmark) were coated with antigens at 1.0 μg/ml (GLURP R2 and MSP1 hybrid) or 5.0 μg/ml (AS202.11) in 1X PBS (pH 7.04). Coated plates were kept in a refrigerator at 2–8 °C overnight. Plates were blocked (PBS with 5 % milk powder, 0.1 % Tween-20) and incubated at room temperature (RT) in a humidified chamber for 1 h. Plasma samples diluted at 1:200 in serum dilution buffer (PBS with 2.5 % milk powder, 0.1 % Tween-20 and 0.02 % Na-azide) were added in duplicates and incubated 2 h at RT. To control for inter-assay and day-to-day variations each ELISA plate included a reference curve obtained by a two-fold titration of pool of hyper immune plasma. In addition, each plate included a negative control sample (a pool of plasma sample from Danish blood donors never exposed to malaria), a positive control sample (plasma from clinically semi-immune adults obtained from the Korle-Bu blood bank, Accra) and a buffer blank. The dilutions for the horseradish peroxidase (HRP) conjugated secondary antibodies used in the assays were: goat anti-human IgG (γ) (H10007) (Invitrogen Corporation, CA, USA) (1:80,000) and goat anti-human IgM (μ) (H15007) (Invitrogen) (1:3000). The IgG sub-classes were detected using HRP-conjugated sheep anti-human IgG1 (AP006) (1:5000), IgG2 (AP007) (1:2000), IgG3 (AP008) (1:10,000) and IgG4 (AP009) (1:1000) antibodies (The Binding Site Group Ltd, UK), respectively. These were added at 100 μl/well to respective plates and incubated for 1 h at RT. Plates were washed (PBS with 0.1 % Tween-20 and 0.5 M NaCl) four times between consecutive steps. Bound secondary antibodies were quantified using TMB (3,3′,5,5′-tetramethylbenzidine) substrate (Kem-En-Tec Diagnosis A/S, Taastrup, Denmark) and incubated in the dark for 30 min. Optical density (OD) was read at 450 nm. The OD values for the test samples were converted into antibody units (AU) with the standard reference curves generated for each ELISA plate using a four-parameter curve-fit Microsoft Excel-based application (ADAMSEL b040, Ed Remark© 2009). Samples were re-tested if the coefficient of variation between duplicate absorbance values were higher than 15 % and plates were also re-tested if the R^2^ value of the standard curve was less than 97 %. The same ELISA protocols, reagents lot and batch of positive and negative control samples were used at both study sites.

### Statistical analysis

Individuals were categorized as either responders or non-responders for each antigen based on IgG cut-off value calculated by the mixture model [32]. Mixture models have been reported extensively in characterising individual’s exposure status in infectious diseases [33–35] and have been applied in malaria research [36–38]. Briefly, normalized (log 10 transformation) IgG AU for each antigen was fitted as the sum of two Gaussian distributions by maximum likelihood methods. The cut-off for responder was defined as mean AU of the narrow distribution (non-responder population) plus three standard deviations. The AS202.11 antigen, IgG sub-class levels were too low in both cohorts during protocol optimization and hence these were not measured in the final ELISA analysis. For each antigen, scatter plot and univariate linear regression analyses were used to assess the relationship between antibody levels and age. Cumulative malaria incidence and 95 % confidence interval estimated by the Kaplan–Meier method were defined as the proportion of children with at least one episode of clinical malaria by the end of surveillance period. Incidence rate was defined as the ratio of the total number of clinical malaria episodes to the total child months at risk. For estimating 95 % confidence interval on the incidence rates, standard errors were calculated using the method described by Stukel et al. [39], which allows for repeated episodes in the same child. This assumes rates are constant over time. Cox regression including all episodes of clinical malaria, and using a robust estimate of the standard error that takes into account the correlation among multiple episodes per person was used to estimate the rate ratio. Antibody level was modelled as continuous variable transformed to log base 10 so that rate ratios indicate the decrease in incidence rate of malaria corresponding to a ten-fold increase in baseline antibody levels. Age, baseline parasitaemia and gender were considered a potential confounder and were adjusted for in the analysis. Age was modelled as a categorical variable with four levels: 6–23, 24–35, 36–47, and 48–72 months. Cases occurring within 14 days of the start of treatment for malaria were assumed to be relapse of the original case and were not counted. The time at risk for each child was calculated based on date of start of follow-up and date of end of the study or until the child was censored. Both site specific and pooled analysis of hazard ratios for the association between antibody levels and clinical malaria were performed. Stata version 10 for Windows (College Station, Texas, USA) and R v3.2.2 [40] were used for the analyses.

## Results

### *Plasmodium falciparum* infection in the study cohorts

Altogether, 563 children aged 6–72 months were included in the study. They comprised 354 and 209 children from Burkina Faso and Ghana, respectively, who completed the entire duration of the follow-up at each site. The Burkinabe children (mean age = 34.2 months) were significantly younger than the Ghanaian children (mean age = 51.8 months) (*p* < 0.0001, Welch Two Sample t-test). No child used insecticide-treated nets (ITN) in the Burkinabe cohort while among the Ghanaian children, ITN usage was 31.6 % but was not associated with clinical malaria (*p* > 0.05, Pearson’s Chi squared test). Baseline parasite prevalence was higher in the Burkinabe, [61.9 %, (219/354)] than in the Ghanaian [2.9 %, (6/209)] cohort (Table 1). The cumulative incidence of malaria decreased with age of study participants, although this observation was more prominent in the Burkinabe than in the Ghanaian cohort (Table 1). Similarly, cumulative malaria incidence was lower in responders to GLURP R2 and MSP1 hybrid in Burkina Faso but this trend was not seen in the Ghanaian cohort. Malaria incidence among responders and non-responders to AS202.11 did not differ either among Burkinabe or Ghanaian children (Table 1). The proportion of children with at least one episode of clinical malaria at the end of the follow-up period was 70.7 % (95 % CI 65.8–75.3) in Burkina Faso and 8.6 % (95 % CI 5.5–13.3) in Ghana (Table 1). The number of malaria episodes per child ranged from nought to seven in Burkina Faso and nought to two in Ghana (Additional file 1: Table S1). A total of 154 (43.5 %) Burkinabe children experienced at least two malaria episodes while only a single child in Ghana had exactly two episodes (Additional file 1: Table S1).

**Table 1:** Characteristics of Burkina Faso and Ghana study populations

Characteristics	Burkina Faso					Ghana				
	N	Cumulative incidence (95 % CI)^a^	Child-months at risk	Malaria cases	Rate per 100 child-months (95 % CI)^b^	N	Cumulative incidence (95 %CI)^a^	Child-months at risk	Malaria cases	Rate per 100 child-months (95 % CI)^c^
Age group (months)										
6–23	99	83.7 % (75.7, 90.2)	1071.2	191	17.8 (15.9, 19.7)	16	43.8 % (23.8, 70.5)	152.7	7	4.6 (2.2, 9.6)
24–35	87	82.5 % (73.7, 89.7)	947.0	176	18.6 (16.6, 20.6)	54	5.6 % (1.8, 16.2)	509.8	3	0.6 (0.2, 1.8)
36–47	90	69.7 % (60, 78.8)	982.0	120	12.2 (10, 14.4)	54	5.6 % (1.8, 16.2)	510.8	3	0.6 (0.2, 1.8)
48–72	78	42.4 % (32.3, 54.2)	851.0	52	6.1 (3.8, 8.4)	85	5.9 % (2.5, 13.6)	802.1	6	0.7 (0.3, 1.7)
Baseline parasitaemia status										
Negative	132	82.6 % (75.7, 88.5)	1432.9	249	17.4 (15.8, 19)	203	8.9 % (5.7, 13.7)	1919.0	19	1.0 (0.6, 1.6)
Positive	219	63.8 % (57.4, 70.2)	2386.5	288	12.1 (10.6, 13.6)	6	0 %	0	0	–
GLURP R2 IgG serological status										
Non-responder	230	80 % (74.6, 84.9)	2499.5	404	16.2 (14.9, 17.4)	167	9 % (5.5, 14.5)	1577.0	16	1.0 (0.6, 1.7)
Responder	124	52.6 % (44, 61.7)	1351.7	135	10 (7.9, 12.1)	42	7 % (2.4, 20.5)	398.3	3	0.8 (0.2, 2.3)
MSP1 hybrid IgG serological status										
Non-responder	272	75.1 % (69.8, 80.2)	2953.8	448	15.2 (13.9, 16.4)	166	8.4 % (5.1, 13.8)	1567.7	15	1.0 (0.6, 1.6)
Responder	82	56.1 % (45.8, 67)	897.4	91	10.1 (7.7, 12.6)	43	9.3 % (3.6, 22.9)	407.6	4	1.0 (0.4, 2.6)
AS202.11 IgG serological status										
Non-responder	313	71 % (65.9, 75.9)	3406.2	486	14.3 (13.1, 15.5)	184	9.2 % (5.9, 14.4)	1738.5	18	1.0 (0.7, 1.6)
Responder	41	68.1 % (53.4, 81.9)	444.9	53	11.9 (9, 14.8)	25	4 % (0.6, 25.2)	236.8	1	0.4 (0.1, 3.0)
Total	354	70.7 % (65.8, 75.3)	3851.2	539	14 (12.9, 15.1)	209	8.6 % (5.5, 13.3)	1975.3	19	1.0 (0.6, 1.5)

*N* number of children

^a^Cumulative incidence and 95 % CI was calculated using Kaplan-Meier method

^b^95 % CI was calculated using the method by Stukel et al., [32]

^c^95 % CI was calculated using standard method for rate; there was only one multiple episode

### Relationship between antibody levels and age of study participants

**Fig. 2:**
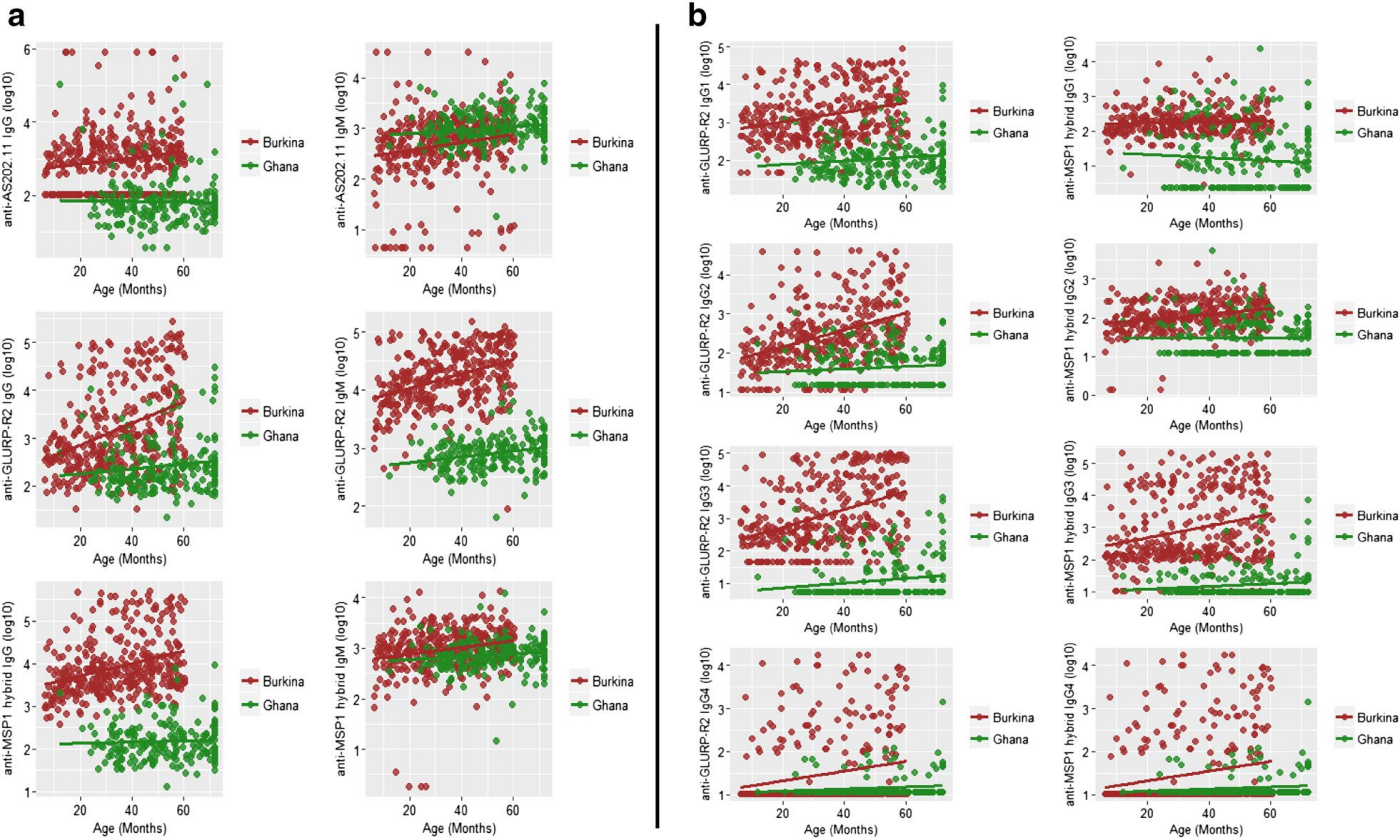
Isotype IgG and IgM and IgG sub-class levels in relation to age. **a** Total IgG or IgM levels against the antigens AS202.11 (*top*), GLURP R2 (*middle*) and MSP1 hybrid (*bottom*), respectively. **b** IgG1, IgG2, IgG3, or IgG4 levels against the antigens GLURP R2 (*left*) and MSP1 hybrid (*right*), respectively. Linear regression lines are shown for each antigen specific antibody level for each site

The levels of isotype IgG, IgM and IgG sub-classes against all three antigens (GLURP R2, MSP1 hybrid and AS202.11), except IgG1 and IgG4 against MSP1 hybrid, increased significantly (*p* < 0.05) with age in children from Burkina Faso (Table 2). Among the Ghanaian children, only levels IgG3 against GLURP R2 and IgM against all the antigens increased significantly (*p* < 0.05) with age. The adjusted R^2^ values from the univariate linear regression analysis of antibody levels and age was generally low especially for the Ghanaian cohort (adjusted R^2^ range: Burkina Faso = 0.0013–0.16; Ghana = 0–0.054) (Table 2). Except for anti-AS202.11 IgM antibody levels, children in the Burkinabe cohort always had higher antibody levels against the studied antigens than those in the Ghanaian cohort (Fig. 2a, b).

**Table 2:** Relationship between antibody levels and age

Burkina Faso				Ghana			
Antibody	Antigen	Coefficient (95 % CI)	*p* value	Adjusted R^2^	Coefficient (95 % CI)	*p* value	Adjusted R^2^
IgG	GLURP R2	6.69 (4.10, 10.91)	<0.0001	0.14	2.82 (0.97, 8.23)	0.057	0.013
	MSP1-hybrid	9.28 (4.75, 18.13)	<0.0001	0.11	1.56 (4.10, 5.93)	0.51	0
	AS202.11	2.23 (1.33, 4.28)	0.0037	0.021	0.86 (0.35, 2.14)	0.73	0
IgG1	GLURP R2	5.55 (2.91, 10.60)	<0.0001	0.069	3.09 (1.00, 9.61)	0.051	0.014
	MSP1-hybrid	2.08 (0.63, 6.83)	0.23	0.0013	0.71 (0.37, 1.36)	0.3	0.0004
IgG2	GLURP R2	10.37 (6.00, 17.94)	<0.0001	0.16	2.39 (0.70, 8.12)	0.16	0.0047
	MSP1-hybrid	21.88 (6.98, 68.57)	<0.0001	0.072	1.00 (0.28, 3.66)	1	0
IgG3	GLURP R2	6.16 (4.02, 9.43)	<0.0001	0.16	2.93 (1.20, 7.16)	0.019	0.021
	MSP1-hybrid	2.81 (1.85, 4.26)	<0.0001	0.061	3.09 (0.90, 1.06)	0.073	0.011
IgG4	GLURP R2	2.76 (1.62, 4.71)	0.00021	0.036	8.81 (0.94, 82.24)	0.056	0.013
	MSP1-hybrid	1.88 (0.96, 3.68)	0.064	0.0069	3.42 (0.62, 18.77)	0.16	0.005
IgM	GLURP R2	36.64 (14.83, 90.52)	<0.0001	0.15	35 (5.01, 248.77)	0.0004	0.054
	MSP1-hybrid	8.76 (3.18, 24.12)	<0.0001	0.045	7.62 (1.05, 55.45)	0.045	0.015
	AS202.11	3.60 (1.75, 7.41)	0.00055	0.031	6.86 (1.20, 3.93)	0.031	0.018

Coefficient, 95 % confidence level (CI), *p* values and adjusted R^2^ values were calculated from univariate linear regression models

### Antigen-specific antibodies in relation to risk of clinical malaria

In the Burkinabe cohort, isotype IgG responses against GLURP R2 (HR 0.84; 95 % CI 0.73–0.97; *p* = 0.01) and MSP1 hybrid (HR 0.83; 95 % CI 0.70–0.98; *p* = 0.03) were significantly associated with reduced risk of clinical malaria after adjusting for age (Table 3). Of the IgG sub-class responses, IgG2 and IgG4 against GLURP R2 and IgG3 against both GLURP R2 and MSP1 hybrid were all significantly associated with reduced risk of malaria (*p* < 0.05) in children from Burkina Faso (Table 3). Isotype IgM responses against both GLURP R2 (HR 0.77; 95 % CI 0.63–0.95; *p* = 0.01) and MSP1 hybrid (HR 0.82; 95 % CI 0.71–0.94; *p* = 0.01) were also significantly associated with reduced risk of clinical malaria in the Burkinabe cohort. In the Ghanaian cohort, only IgG3 against GLURP R2 was significantly (HR 0.54; 95 % CI 0.30–0.98; *p* = 0.04) associated with decreased risk of malaria (Table 3). When the two cohorts were combined, all GLURP R2 antibodies, except IgG1, were significantly associated with reduced risk of malaria as was IgM against MSP1 hybrid (Table 3). In a final model to assess immunological variables independently associated with malaria, IgG3 against GLURP R2 (in both study sites) and IgM against MSP1 hybrid (in Burkina Faso only) were significantly (*p* < 0.05) associated with reduced risk of malaria (Table 4). On the other hand, IgG against AS202.11 was associated with increased risk of clinical malaria (HR 1.29; 95 % CI 1.01–1.65; *p* = 0.04) in the Ghanaian cohort (Table 4).

**Table 3:** Association between antibody levels and clinical malaria among children from Burkina Faso and Ghana

Antibody	Antigens	Burkina Faso			Ghana			Overall		
		Crude HR (95 % CI)	HR adjusted for age (95 % CI)^a^	*p* value for adjusted HR	Crude HR (95 % CI)	HR adjusted for age (95 % CI)^a^	*p* value for adjusted HR	Crude HR (95 % CI)^b^	HR adjusted for age (95 % CI)^b^	*p* value for adjusted HR
IgG	GLURP R2	0.75 (0.66, 0.85)	0.84 (0.73, 0.97)	0.01	0.77 (0.44, 1.34)	0.73 (0.41, 1.30)	0.28	0.75 (0.66, 0.85)	0.85 (0.74, 0.97)	0.02
	MSP1-hybrid	0.73 (0.61, 0.87)	0.83 (0.70, 0.98)	0.03	1.40 (0.64, 3.08)	1.19 (0.57, 2.50)	0.64	0.75 (0.63, 0.89)	0.85 (0.72, 1.01)	0.06
	AS202.11	0.93 (0.82, 1.05)	0.97 (0.87, 1.09)	0.65	1.37 (0.89, 2.11)	1.15 (0.85, 1.56)	0.38	0.93 (0.82, 1.06)	0.98 (0.88, 1.10)	0.76
IgG1	GLURP R2	0.86 (0.73, 1.00)	0.97 (0.82, 1.14)	0.68	0.93 (0.47, 1.85)	0.91 (0.45, 1.86)	0.8	0.86 (0.74, 1.00)	0.97 (0.83, 1.14)	0.69
	MSP1-hybrid	0.96 (0.75, 1.23)	0.96 (0.77, 1.21)	0.76	1.22 (0.78, 1.92)	1.03 (0.67, 1.58)	0.91	1.00 (0.80, 1.25)	1.01 (0.82, 1.24)	0.94
IgG2	GLURP R2	0.74 (0.65, 0.83)	0.85 (0.75, 0.96)	0.01	1.45 (0.71, 2.97)	1.24 (0.64, 2.43)	0.52	0.74 (0.66,0.84)	0.86 (0.76, 0.98)	0.02
	MSP1-hybrid	0.73 (0.59, 0.91)	0.87 (0.70, 1.08)	0.2	1.19 (0.47, 3.01)	1.17 (0.51, 2.67)	0.72	0.74 (0.60, 0.92)	0.89 (0.72, 1.10)	0.27
IgG3	GLURP R2	0.73 (0.66, 0.81)	0.81 (0.73, 0.90)	<0.0001	0.48 (0.25, 0.93)	0.54 (0.30, 0.98)	0.04	0.73 (0.66, 0.81)	0.81 (0.73, 0.90)	<0.0001
	MSP1-hybrid	0.85 (0.78, 0.93)	0.91 (0.84, 0.99)	0.04	0.82 (0.38, 1.77)	0.83 (0.33, 2.08)	0.69	0.86 (0.78, 0.94)	0.92 (0.84, 1.00)	0.05
IgG4	GLURP R2	0.79 (0.68, 0.92)	0.85 (0.73, 0.98)	0.02	1.18 (0.31, 4.57)	1.30 (0.38, 4.43)	0.68	0.79 (0.68, 0.92)	0.85 (0.73, 0.98)	0.02
	MSP1-hybrid	0.95 (0.83, 1.09)	0.97 (0.85, 1.11)	0.65	1.14 (0.32, 4.03)	0.82 (0.29, 2.31)	0.7	0.95 (0.83, 1.09)	0.98 (0.86, 1.11)	0.73
IgM	GLURP R2	0.66 (0.55, 0.79)	0.77 (0.63, 0.95)	0.01	0.70 (0.14, 3.57)	1.14 (0.24, 5.40)	0.87	0.66 (0.55, 0.79)	0.78 (0.64, 0.95)	0.02
	MSP1-hybrid	0.72 (0.63, 0.84)	0.81 (0.70, 0.94)	0.01	1.42 (0.25, 8.07)	1.68 (0.36, 7.93)	0.51	0.73 (0.63, 0.84)	0.82 (0.70, 0.95)	0.01
	AS202.11	0.86 (0.74, 0.99)	0.92 (0.80, 1.05)	0.23	0.58 (0.23, 1.45)	0.70 (0.26, 1.90)	0.49	0.85 (0.74, 0.98)	0.92 (0.80, 1.06)	0.23

Crude hazard ratios (HR) as well as age-adjusted HR and *p* values are shown, indicating the ratio of malaria hazard rates associated with a tenfold increase in baseline antibody level

^**a**^95 % CI were estimated using Cox regression model with robust standard error to adjust for clustering in children

^**b**^Stratified estimates (i.e., equal coefficients across sites but with baseline hazard unique to each site)

**Table 4:** Adjusted hazard ratios for immunological variables independently associated with malaria risk in the final model

Sites	Immunological/age variables	Adjusted HR (95 % CI)	Wald *p* value
Burkina Faso	GLURP R2 IgG3 (values transformed to log base 10)	0.82 (0.74, 0.91)	*p* < 0.0001
	MSP1-hybrid IgM (values transformed to log base 10)	0.85 (0.73, 0.98)	*p* = 0.02
	Age group (months)		
	6–23	1	*p* < 0.0001
	24–35	1.12 (0.91, 1.38)	
	36–47	0.82 (0.62, 1.07)	
	48–60	0.44 (0.31, 0.63)	
Ghana	GLURP R2 IgG3 (values transformed to log base 10)	0.48 (0.25, 0.91)	*p* = 0.02
	AS202.11 IgG (values transformed to log base 10)	1.29 (1.01, 1.65)	*p* = 0.04
	Age group (months)		
	6–23	1	*p* < 0.01
	24–35	0.14 (0.04, 0.48)	
	36–47	0.14 (0.04, 0.48)	
	48–72	0.19 (0.07, 0.55)	

Hazard ratios (HR) indicates the ratio of malaria hazard rates associated with a ten-fold increase in baseline antibody level

## Discussion

The association between antibody levels against GLURP R2, MSP1 block 2 hybrid and AS202.11 and the risk of clinical malaria was investigated in two independent immune-epidemiological studies conducted in malaria hyperendemic (Burkina Faso) and hypo-endemic (Ghana) populations, respectively, using the same study protocols and reagents lots. Overall, high levels of IgG3 against GLURP R2 was strongly associated with reduced risk of clinical malaria in both Burkinabe and Ghanaian children despite the marked differences in malaria transmission intensity between these two populations. In Burkinabe children, IgM responses against MSP1 hybrid contributed significantly to reducing the risk of clinical malaria while in Ghanaian children, increased levels of IgG against the peptide AS202.11 was associated with about 29 % increased risk of clinical malaria (Table 4). In previous studies, IgG against AS202.11 was found to increase with age and associated with protection from malaria of Kenyan children [25] living in an area of high malaria transmission (EIR ranging from 20 to 100 infective bites per year [41]). Here, malaria incidence in the Burkinabe cohort was very high, and although AS202.11 IgG and IgM antibodies clearly increased with age, none was associated with protection against malaria. In addition, the weak IgG responses to AS202.11 in the malaria hypo-endemic population of Ghana, with its subsequent association with increased malaria risk, suggests it may rather be a marker of exposure in the studied populations.

In most malaria-endemic populations, intensified transmission prevention measures such as use of ITNs, indoor residual spraying and effective therapeutics are rapidly changing the landscape of malaria epidemiology [42, 43]. Malaria incidence in the Burkinabe cohort was more than eight-fold higher than in the Ghanaian cohort possibly due to the lack of ITN usage in the former. It may also be due to the risk of malaria being higher in younger children living in communities where transmission intensity is high [1, 44, 45]. Thus, the Ghanaian cohort consisting of older children living in a malaria hypo-endemic region was already at a much lower risk of getting malaria. In populations with steady and high malaria transmission intensity, acquired immunity to clinical malaria develops rapidly with frequency of exposure to the parasite and age [45, 46]. This may be due to acquisition of a broad spectrum of protective antibodies in such populations early in life. High intensity of transmission may also be necessary for sustaining malaria-specific antibodies at high levels and functionality [45, 47]. Conversely, with a decline in malaria transmission intensity, as was evident in the Ghanaian cohort, prolonged exposure over relatively longer periods may be necessary in building a sufficient repertoire of protective antibodies [45]. This may explain why antibody associations with protection were more distinct in the Burkinabe children than in the Ghanaian children. In the antibody association with age models, the low R^2^ values observed, particularly for the Ghanaian cohort, suggests in areas of very low malaria transmission, age alone may not adequately explain acquisition of malaria-specific antibodies.

In previous studies, GLURP R2 IgG was associated with protection against malaria in Burkinabe [20] and Myanmar [48] children. The present study supports these finding since high titres of IgG antibodies to GLURP R2 were significantly associated with reduced risk of malaria with IgG3 showing the strongest effect in both cohorts. The association of GLURP R2 IgG2 titres with reduced risk of malaria in the Burkinabe cohort may be due to the observation that IgG2 binds with high affinity to Fc gamma receptor IIA-131H (FcγRIIA-131H) allele on immune cells of individuals expressing this variant [49–51]. Although FcγRIIA was not genotyped in the present study, other studies in the same district in Burkina Faso reported FcγRIIA-131H-allele frequency to be 28.6 % in the population [52]. Thus, individuals with this FcγRIIA-131H polymorphism could benefit from GLURP R2 IgG2 mediated cellular effector functions that may afford protection against malaria. MSP1 block 2 epitopes have been shown to be important targets of antibodies associated with protection from clinical malaria [6, 13, 14]. *Plasmodium falciparum* diversity at the Ghanaian site is unknown, however, the K1 and MAD 20 allelic families of *msp1* have been shown to be the most prevalent in Balonghin, Burkina-Faso [53]. Here, the MSP1 hybrid used was specifically designed to incorporate most of the block 2 allelic variants purported to be targets of protective antibodies against malaria [8]. Moreover, the present study is the first to show antibody responses against the MSP1 block 2 hybrid to be associated with reduction in risk of symptomatic *P. falciparum* infections. Both GLURP R2 and MSP1 block 2 have been shown to elicit antibodies capable of inhibiting parasite growth *in vitro* in the monocyte-dependent mechanism, antibody-dependent cellular inhibition (ADCI) [19, 54]. This may explain the strong association with protection observed with IgG3 antibodies to these antigens since IgG3 is considered superior to IgG1 in parasite clearance [55]. Antigen-specific IgM antibody responses are usually associated with primary infection and not so commonly with protection from disease. However, previous studies in both Ghana [16] and Burkina Faso [56] have found association with protection and IgM antibodies to MSP1 and GLURP. In the current study, IgM against MSP1 hybrid was associated with reduced risk of malaria in Burkinabe children, supporting the view that the function of IgM may complement IgG in anti-malarial immunity [57]. Although anti-MSP1 hybrid IgM levels were similar in both cohorts, the association with protection was only seen in the Burkina Faso cohort. This may reflect differences in epitope specificity and/or avidity of IgM responses under the different malaria transmission intensities in the two cohorts. The possible mechanism(s) by which IgM could mediate protection in malaria remains unclear and several hypotheses have been proposed [58]. It has been suggested that IgM with its pentameric structure capable of binding ten antigenic sites may play a role in blocking merozoite invasion of erythrocytes through agglutination or may mediate antibody-dependent phagocytic mechanisms [56] or complement-mediated anti-parasitaemic processes [58]. Functional studies investigating the effect of malaria-specific IgM antibodies on parasite growth will be needed to elucidate the possible mechanism by which IgM mediates protection against malaria.

## Conclusion

This study corroborates findings from previous studies associating GLURP R2 as an important target for protective antibodies against malaria. The results show for the first time, that antibodies against the chimeric MSP1 block 2 hybrid are associated with reduced risk of malaria in a hyperendemic population. The findings support the further development of both GLURP R2 and MSP1 block 2 hybrid as important blood stage vaccine candidates that may be deployed in children living in areas of different malaria endemicity. These may be most effective as a single fused vaccine antigen rather than as individual sub-unit vaccines.

## Additional file

10.1186/s12936-016-1146-4 Malaria episodes in Burkinabe and Ghanaian children in the cohorts.

## Abbreviations

GLURP R2: glutamate rich protein region 2
MSP1: merozoite surface protein 1
FcγRIIA: Fc gamma receptor IIA
ITN: insecticide-treated bed net
SNP: single nucleotide polymorphism
EIR: entomological inoculation rate
TTBS: thick and thin film blood slides
Hb: haemoglobin
RT: room temperature
HRP: horseradish peroxidase
OD: optical density
AU: antibody unit
ADCI: antibody dependent cellular inhibition
