# Supplementary material for: Antibody levels against GLURP R2, MSP1 block 2 hybrid and AS202.11 and the risk of malaria in children living in hyperendemic (Burkina Faso) and hypo-endemic (Ghana) areas
Source: Malar J. 2016 Feb 27;15:123. doi: 10.1186/s12936-016-1146-4 (PMC4769494; doi:10.1186/s12936-016-1146-4)
Supplement: Supplementary file 1 — 10.1186/s12936-016-1146-4 Malaria episodes in Burkinabe and Ghanaian children in the cohorts. [file 12936_2016_1146_MOESM1_ESM.doc]

**Table S1 Malaria episodes in Burkinabe and Ghanaian children in the cohorts**

| **Number of episodes** | **Burkina Faso (N=354)** | | **Ghana (N=209)** | |
| --- | --- | --- | --- | --- |
|  | **Number of children** | **%** | **Number of children** | **%** |
| **0** | 103 | 29.1 | 190 | 90.9 |
| **1** | 97 | 27.4 | 18 | 8.6 |
| **2** | 69 | 19.5 | 1 | 0.5 |
| **3** | 46 | 13.0 | 0 | 0 |
| **4** | 26 | 7.3 | 0 | 0 |
| **5+** | 13* | 3.7 | 0 | 0 |

*These 13 children included 10 who had 5 episodes, 2 with 6 episodes and 1 who had 7 episodes.
